# Supplementary figures and images for: Enhanced translation expands the endo-lysosome size and promotes antigen presentation during phagocyte activation
Source: PLoS Biol. 2019 Dec 4;17(12):e3000535. doi: 10.1371/journal.pbio.3000535 (PMC6913987; doi:10.1371/journal.pbio.3000535)

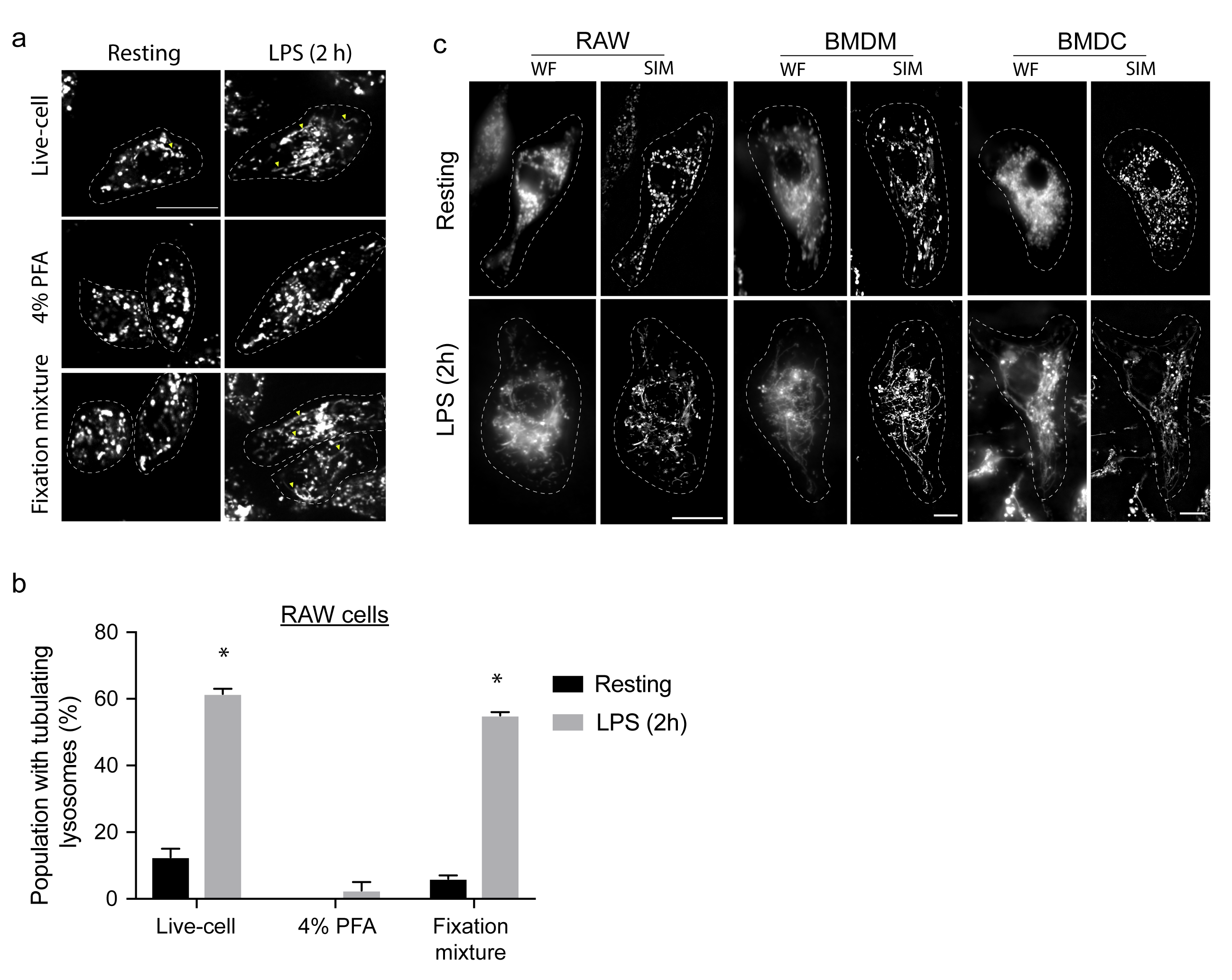

Supplement: S1 Fig — (a) RAW macrophage lysosomes labelled with fluid-phase fluorescent probes were imaged live or fixed with 4% PFA or a mixture of PFA and glutaraldehyde as explained in Materials and methods. (b) Percent lysosome tubulation was recorded within the population for cells exhibiting 4 or more lysosomal tubules longer than 4 μm. Statistical analysis was done with an ANOVA, in which a single asterisk indicates conditions that are statistically distinct from the corresponding resting group (*p < 0.05). (c) WF illumination or SIM images of lysosomes in RAW macrophages, BMDM, and BMDCs before and after 2 h of LPS stimulation. Scale bar = 5 μm. See S9 Data for original data in S1 Fig. BMDC, bone marrow–derived dendritic cell; BMDM, Bone marrow–derived macrophage; SIM, structured illumination microscopy; WF, wide field. (TIF) [file pbio.3000535.s001.tif]

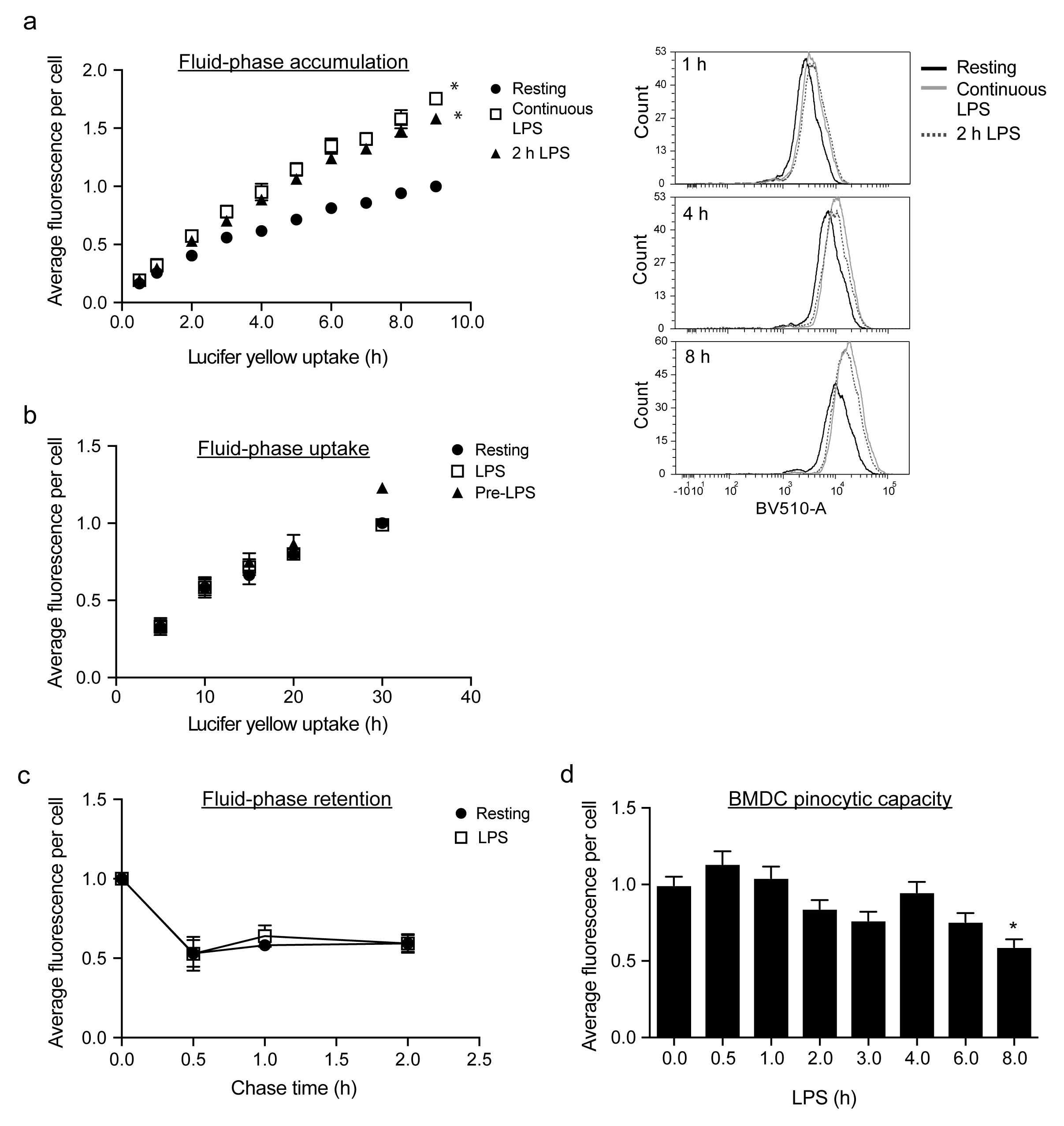

Supplement: S2 Fig — (a) Accumulation of LY in resting and activated RAW macrophages. RAW cells were stimulated and then allowed to internalize LY over time. (b) Pinocytosis rate by quantifying uptake of LY in RAW macrophages treated as indicated. (c) Retention of LY chased in probe-free medium in RAW cells previously treated as indicated and prelabelled with LY for 1 h. In all cases, fluorescence measurements were done by flow cytometry. (d) Pinocytosis in increasingly maturing DCs exposed to LPS. Microscopy was used to measure the uptake of fluorescent dextran for 30 minutes by DCs exposed to LPS over indicated time points. Shown is the mean ± standard error of the mean from at least 3 experiments. For statistical analysis, ANOVA or analysis of covariance was used, in which an asterisk indicates a significant difference in fluorescent probe levels compared to resting (*p < 0.05). See S10 Data for original data in S2 Fig. DC, dendritic cell; LPS, lipopolysaccharides; LY, Lucifer yellow. (TIF) [file pbio.3000535.s002.tif]

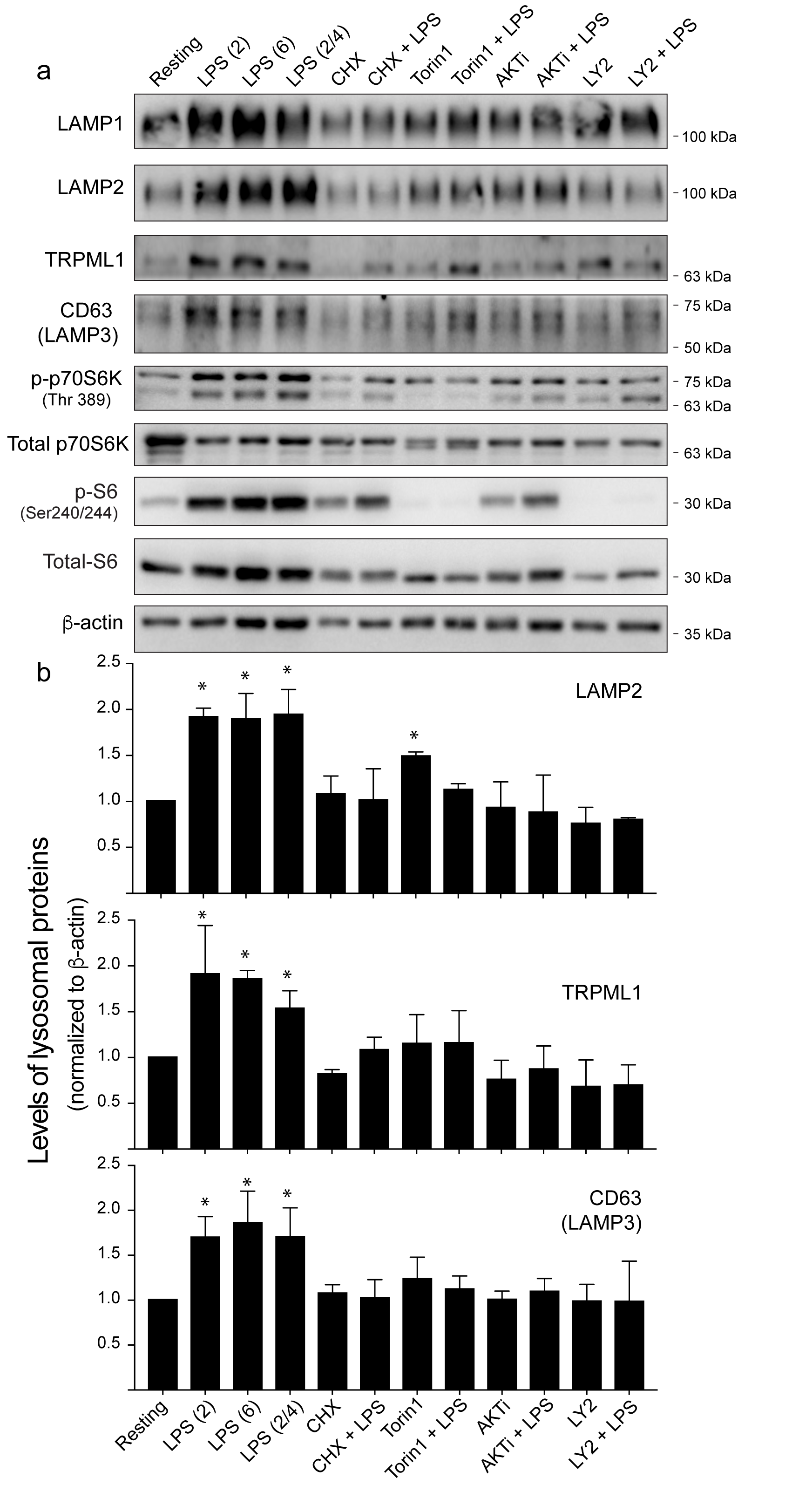

Supplement: S3 Fig — (a) Western blot analysis of additional lysosomal proteins from whole cell lysates of resting primary macrophages or macrophages exposed to the indicated combinations and time of LPS, CHX, Torin1, LY2, AKTi. (b) Quantification of Western blots showing the levels of LAMP2, TRPML1, and CD63 (LAMP3) normalized to actin. Data shown as the mean ± SEM from at least 3 independent experiments. For panels A and B, ‘2/’ indicates cells stimulated with 2 h of LPS, followed by a 4 h chase, whereas ‘2 h’ and ‘6 h’ represent cells continuously exposed to LPS. See S11 Data for original data in S3 Fig. AKTi, AKT inhibitor; CD63, cluster of differentiation protein 63; CHX, cycloheximide; LAMP3, lysosome-associated membrane protein 3; LPS, lipopolysaccharides; LY, Lucifer yellow; LY2, LY2584702; mTOR, mechanistic target of rapamycin; S6K, S6 kinase; TRPML1, transient receptor potential mucolipin 1. (TIF) [file pbio.3000535.s003.tif]

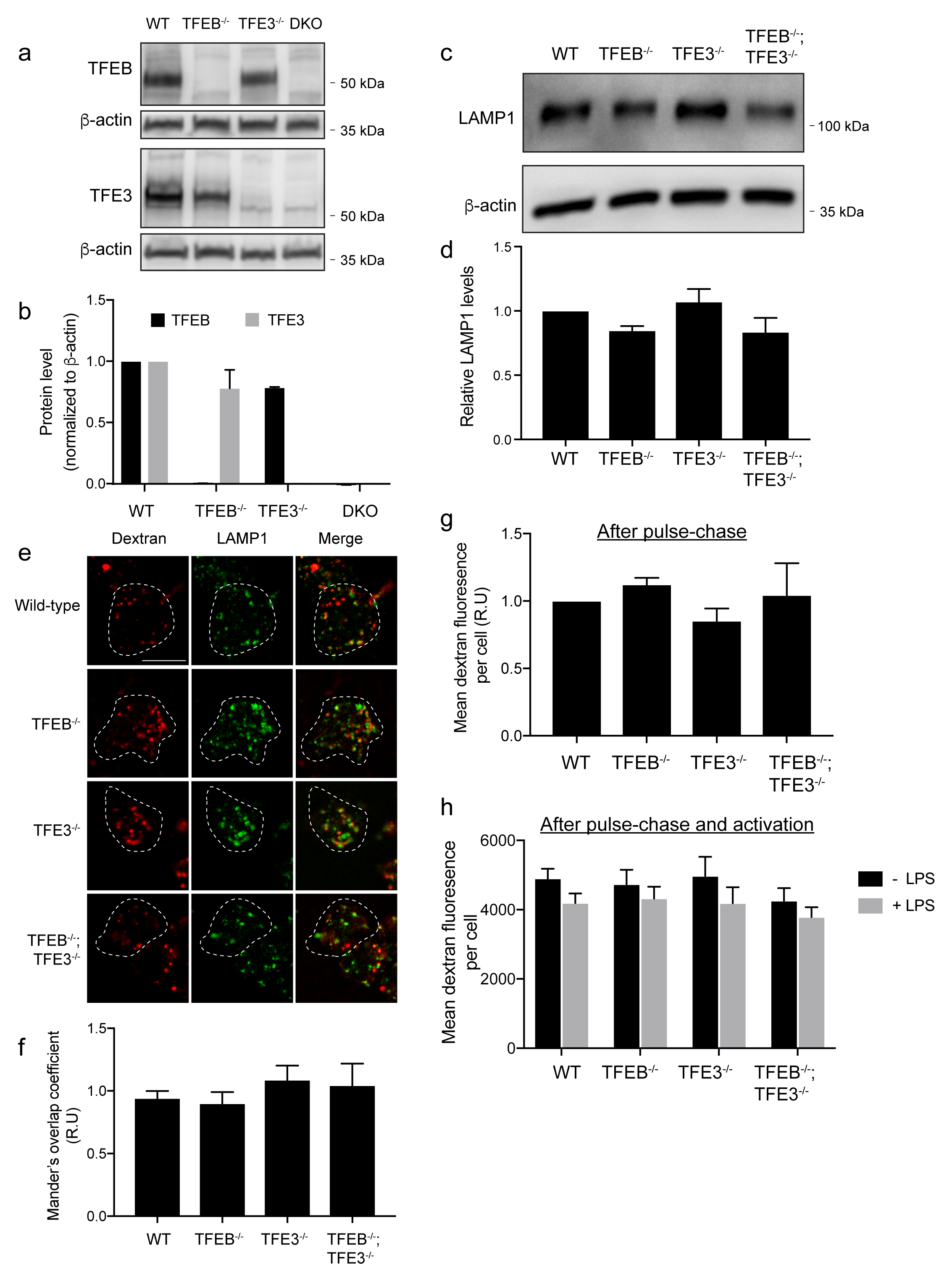

Supplement: S4 Fig — (a–b) Western blot analysis of whole-cell lysates from TFEB−/−, TFE3−/− and double deleted cell lines. (b) Quantification showing mutant lines are devoid of TFEB and/or TFE3 proteins from 3 independent blots. (c) LAMP1 levels in whole-cell lysates from wild-type and deletion mutants of TFEB and/or TFE3. (d) Quantification of LAMP1 levels in knock-out cells. LAMP1 levels were normalized to β-actin to control for loading. Statistical analysis using ANOVA determined that LAMP1 levels did not vary across strains. (e) Colocalization of dextran and LAMP1 in wild-type and deletion strains. Right, middle, and left panels show dextran (red), endogenous LAMP1 (green) and merge, respectively. Scale bar = 5 μm. (f) Mander’s coefficient of dextran co-localizing in LAMP1 structures. Data are shown as RU, normalized to wild-type strain. (g) Pinocytosis label after a 1 h pulse and 1 h chase of fluorescent dextran in resting wild-type and deletion RAW strains, measured by microscopy and image analysis. Mean fluorescence intensity was normalized to wild-type strain and is represented as RU. (h) Dextran fluorescence in RAW and deletion strains 2 h after LPS exposure or vehicle. For all data, shown are the mean± standard deviation from at least 3 independent experiments. See S12 Data for original data in S4 Fig. LAMP1, lysosome-associated membrane protein 1; LPS, lipopolysaccharides; RU, relative units; TFEB, transcription factor EB; TFE3, transcription factor E3. (TIF) [file pbio.3000535.s004.tif]

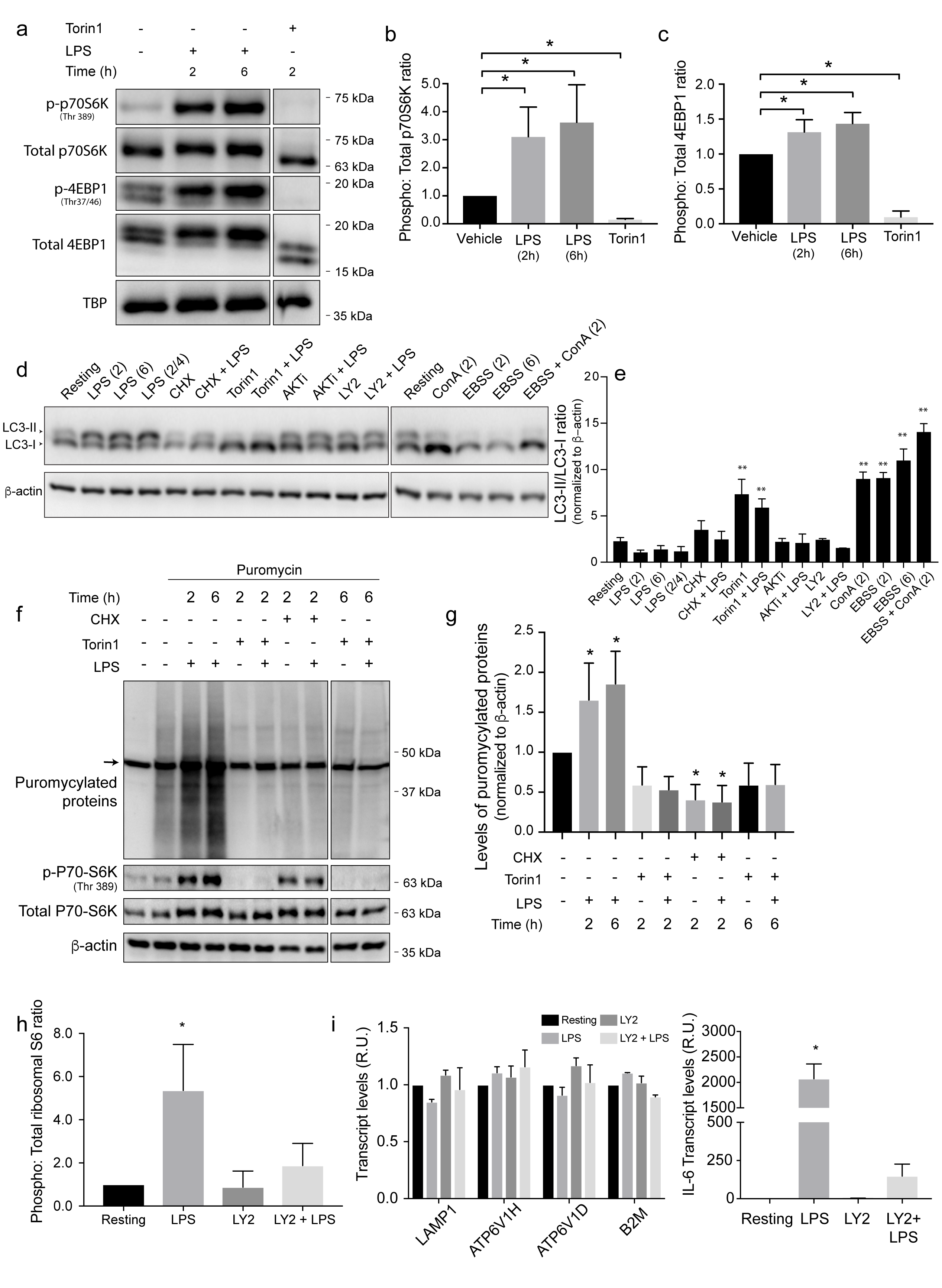

Supplement: S5 Fig — (a) Western blot analysis of whole-cell lysates from resting and activated primary macrophages. Total levels and phosphorylation status of S6K and 4E-BP1 were monitored using the indicated antibodies. TBP served as a loading control. (b–c) Normalized ratio of (b) p-p70S6K and (c) p-4EBP1 to total p70S6K and 4E-BP1 protein. Shown is the mean ± standard deviation from 3 independent blots. (d) Western blot analysis of LC3-I to LC3-II conversion to measure treatment effect on autophagy induction in primary macrophages. BMDMs were activated with LPS in the presence or absence of protein synthesis, mTOR and S6K inhibitors for the time points indicated in brackets. ConA and EBSS treatment was used as a positive control for autophagy induction. (e) Quantification of panel d from 3 independent experiments. Ratio of LC3II to LC3I levels was normalized to actin loading control. (f) Western blot analysis of protein puromycylation in resting and activated primary macrophages. LPS increases the amount of puromycylation indicating a boost in global protein synthesis that is blocked by mTOR inhibitors or cycloheximide. Lane 1 is control lysates from cells not exposed to puromycin. The band indicated by the arrow is a nonspecific band recognized by the anti-puromycin antibody. p-p70S6K and β-actin were used to monitor mTOR status and as a loading control, respectively. (g) Normalized puromycylation signal (excluding nonspecific band) normalized over β-actin signal. Data are shown as the mean ± standard deviation from 4 independent experiments. For panels b, c, e, and g, statistical analysis was done with an ANOVA, in which a asterisk or two asterisks indicates conditions that are statistically distinct from control group (*p < 0.05). (h) Normalized ratio of phosphorylated ribosomal S6 to total ribosomal S6 as depicted in Fig 5F in primary macrophages treated with LY2 alone or co-incubated with LPS for 2 h. Shown is the mean ± standard deviation of the mean from 5 independent blots. [file pbio.3000535.s005.tif]

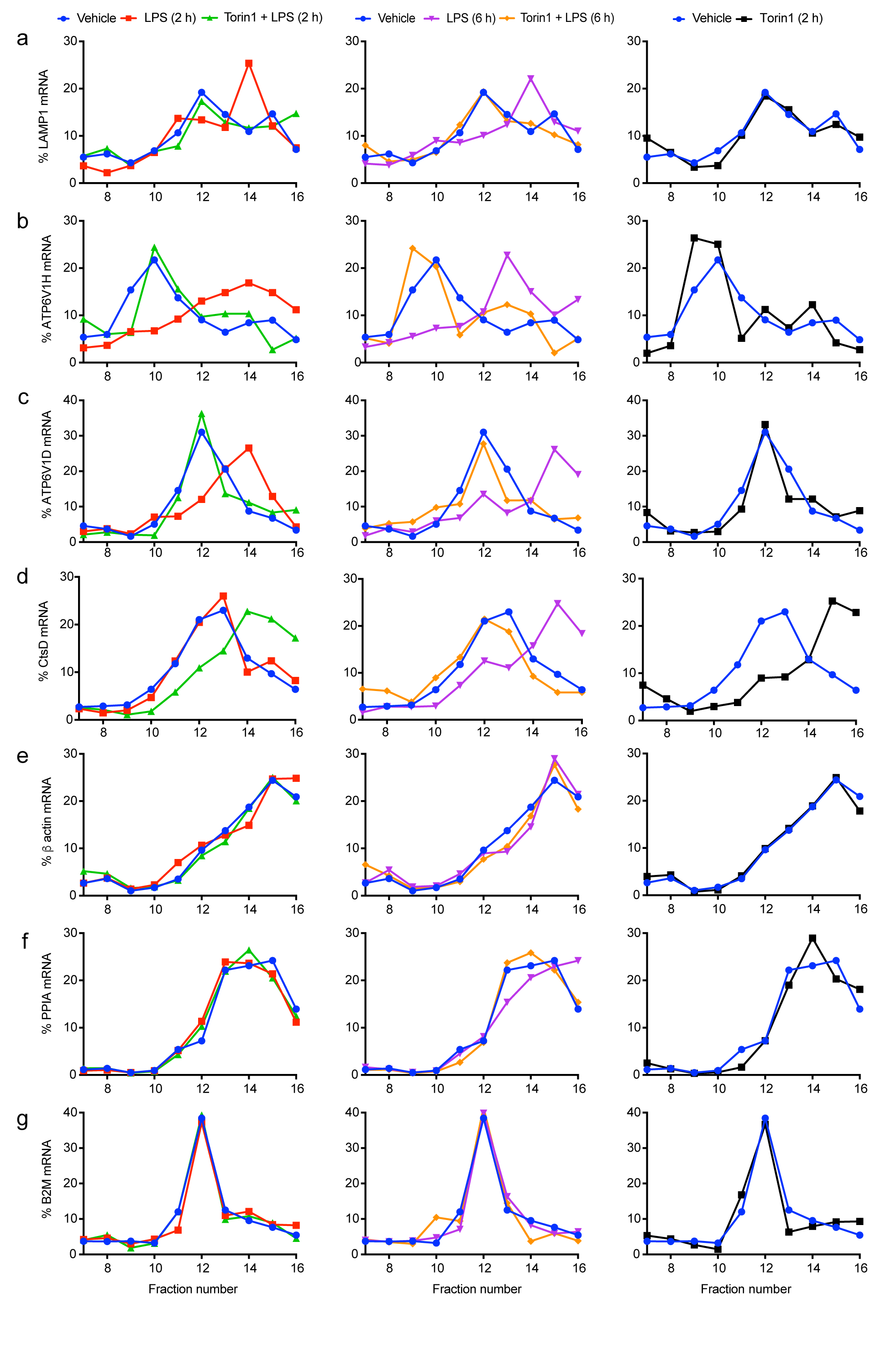

Supplement: S6 Fig — Percent of target mRNA—(a) LAMP1, (b) ATP6V1H, (c) ATP6V1D, (d) CtsD, (e) β-actin, (f) PPIA, and (g) B2M—associated with each ribosome fraction in resting, LPS-treated macrophages, and macrophages co-exposed to LPS and torin1 or treated with torin1 alone. Left, middle, and right panels show 2 h, 6 h, and torin1 (2 h) treatments, respectively. Shown is an additional biological replicate of the experiment described in Fig 7. See S14 Data for original data in S6 Fig. ATP6V1D, V-ATPase V1 subunit D; ATP6V1H, V-ATPase V1 subunit H; B2M, β2-microglobulin; CtsD, Cathepsin D; LAMP1, lysosome-associated membrane protein 1; LPS, lipopolysaccharides; PPIA, peptidylpropyl isomerase A. (TIF) [file pbio.3000535.s006.tif]

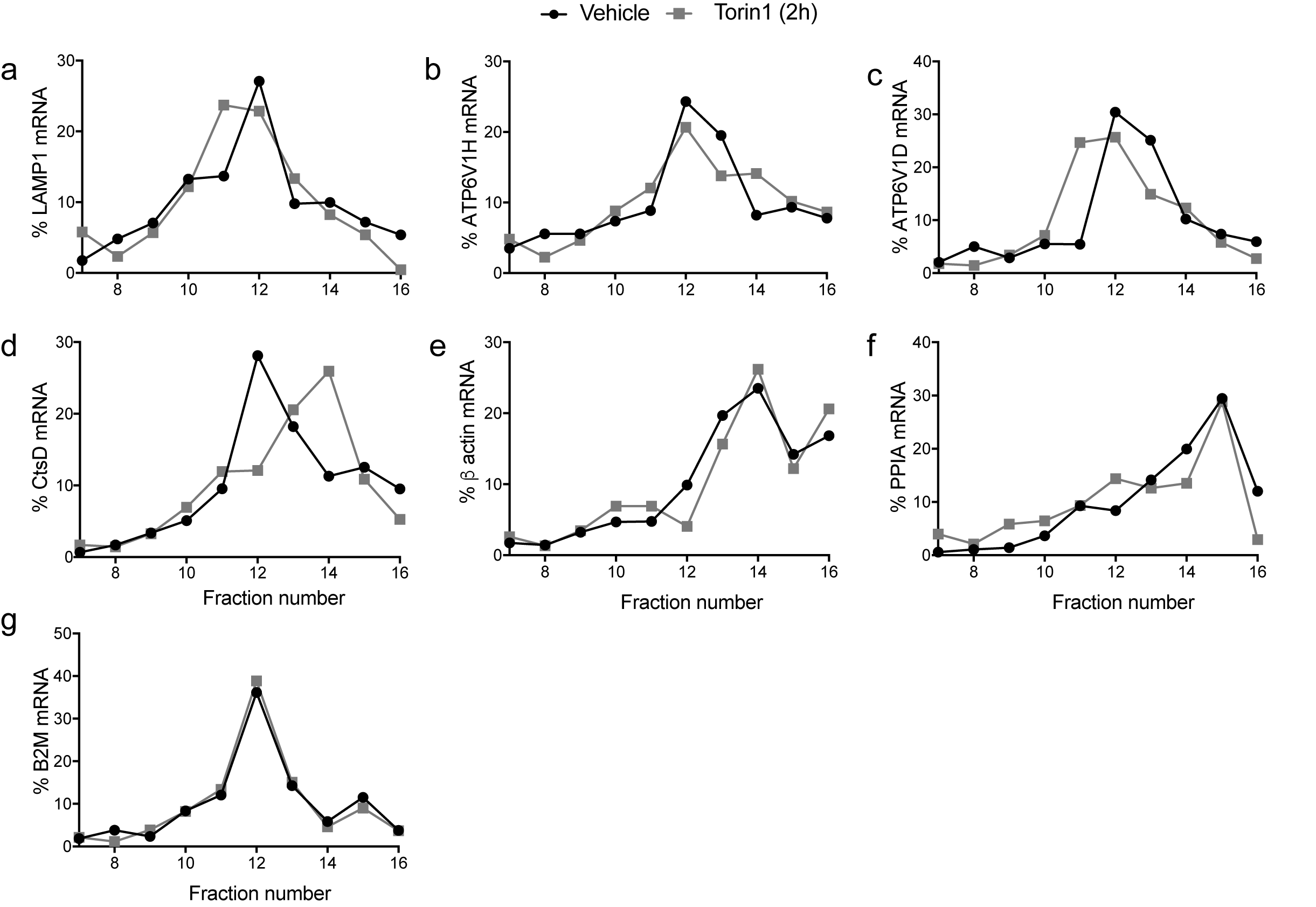

Supplement: S7 Fig — Percent of target mRNA—(a) LAMP1, (b) ATP6V1H, (c) ATP6V1D, (d) CtsD, (e) β-actin, (f) PPIA, and (g) B2M—associated with each ribosome fraction in resting and torin1 (2 h; 100 nM) treated cells for data presented in Fig 7. See S15 Data for original data in S7 Fig. ATP6V1D, V-ATPase V1 subunit D; ATP6V1H, V-ATPase V1 subunit H; B2M, β2-microglobulin; CtsD, Cathepsin D; LAMP1, lysosome-associated membrane protein 1; LPS, lipopolysaccharides; PPIA, peptidylpropyl isomerase A. (TIF) [file pbio.3000535.s007.tif]

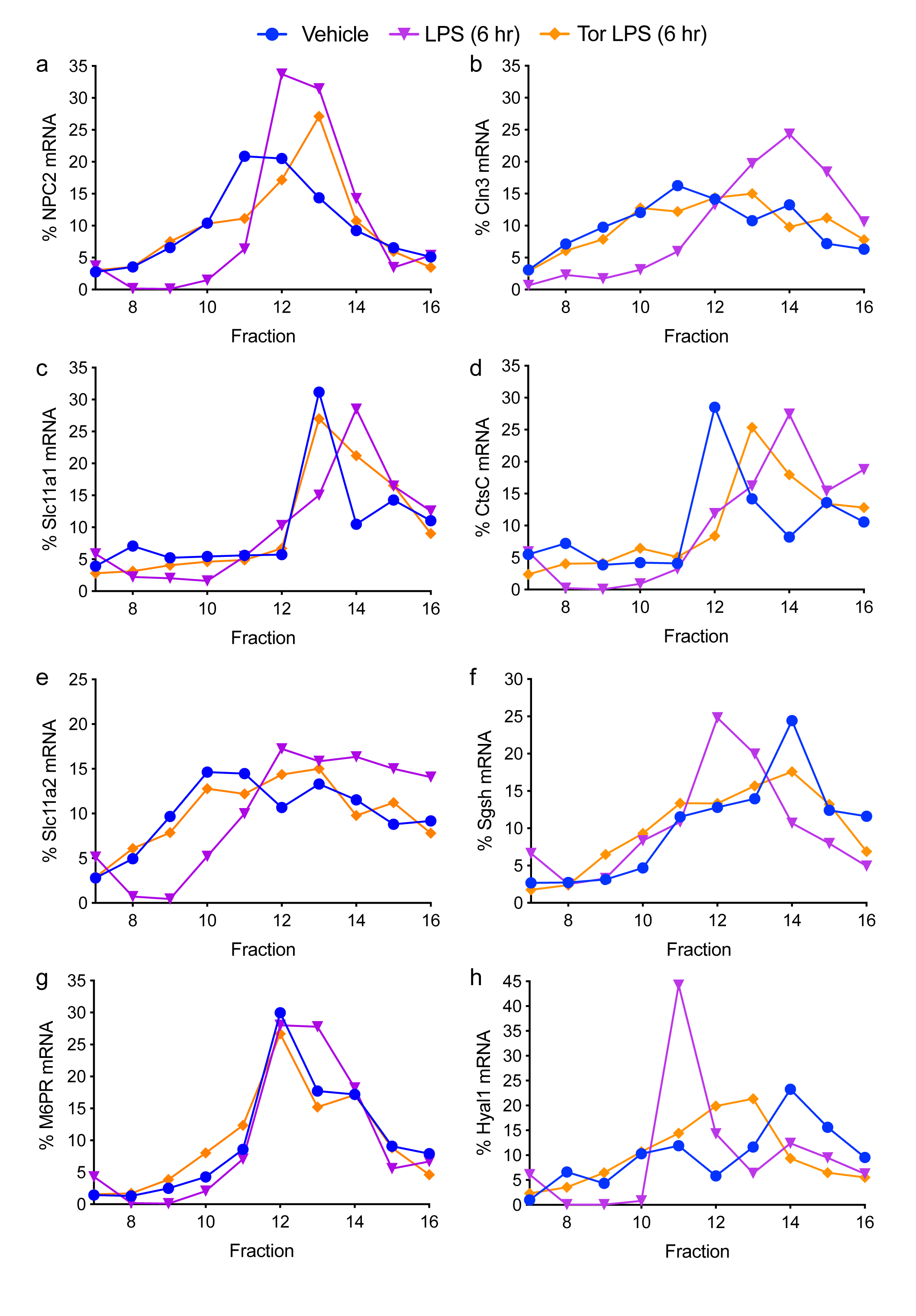

Supplement: S8 Fig — Percent of target mRNA—(a) NPC2, (b) Cln3, (c) Slc11a1, (d) CtsC, (e) Slc11a2, (f) Sgsh, (g) M6PR, and (h) Hyal1—associated with each ribosome fraction in resting, LPS-treated macrophages, and macrophages co-exposed to LPS and torin1. See S16 Data for original data in S8 Fig. Cln3, neuronal ceroid-lipofuscinosis 3; Hyal1, Hyaluronidase-1; LPS, lipopolysaccharides; M6PR, mannose-6-phosphate receptor; NPC2, Niemann-Pick disease type C2 protein; RNAseq, RNA sequencing; Sgsh, sulfamidase; Slc11a1, solute carrier 11A1; Slc11a2, solute carrier 11A2. (TIF) [file pbio.3000535.s008.tif]

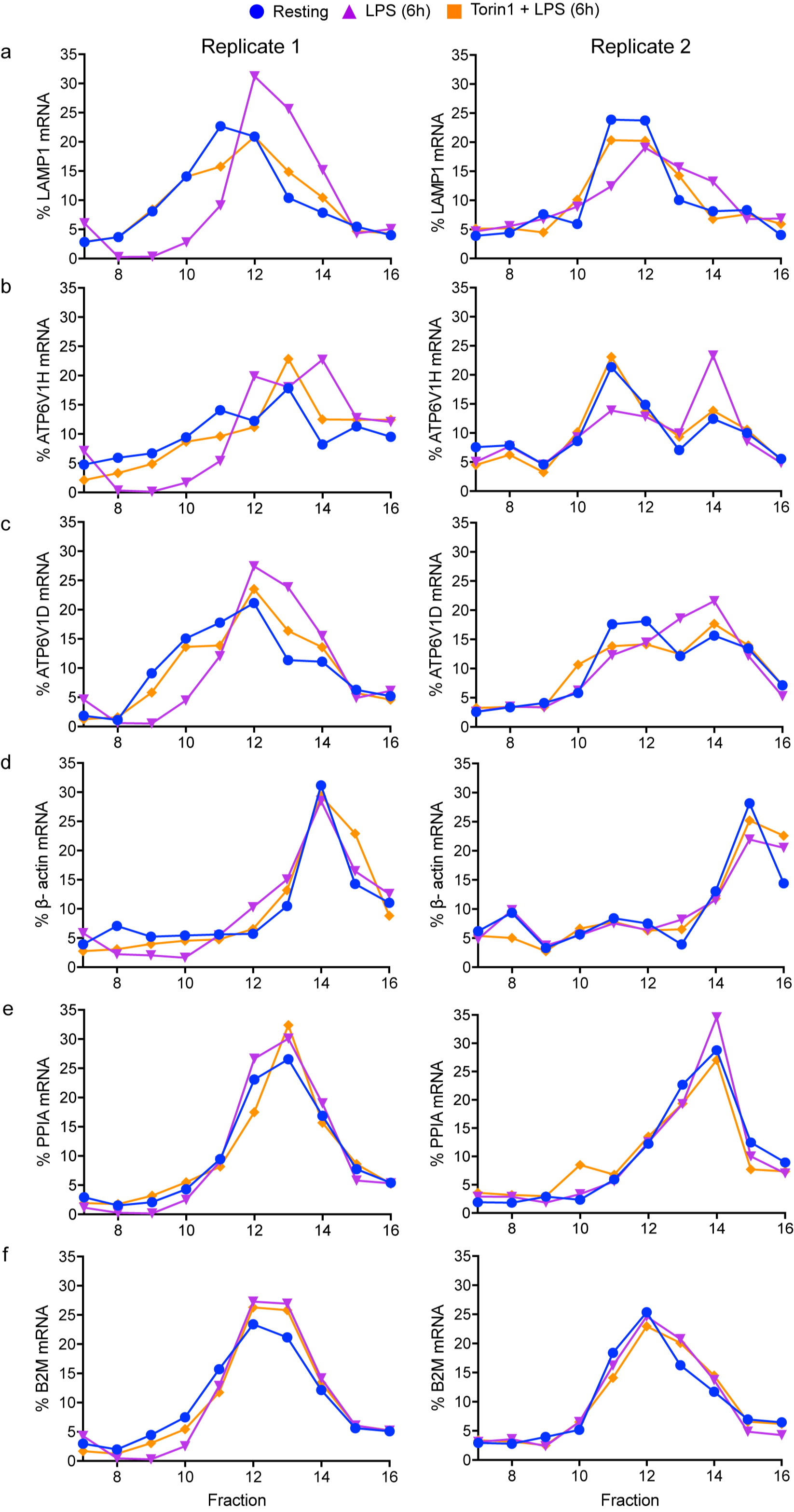

Supplement: S9 Fig — Percent of target mRNA—(a) LAMP1, (b) ATP6V1H, (c) ATP6V1D, (d) β-actin, (e) PPIA, and (f) B2M—associated with each polysome fraction in resting, LPS-treated macrophages and macrophages co-exposed to LPS and torin1 for 6 h. Biological replicate 1 (left) and replicate 2 (right) of data presented in Fig 8 global RNAseq analysis from a total of 3 experiments. See S17 Data for original data in S9 Fig. ATPV1D, V-ATPase V1 subunit D; ATP6V1H, V-ATPase V1 subunit H; B2M, β2-microglobulin; LAMP1, lysosome-associated membrane protein 1; LPS, lipopolysaccharides; PPIA, peptidylpropyl isomerase A; RNAseq, RNA sequencing. (TIF) [file pbio.3000535.s009.tif]

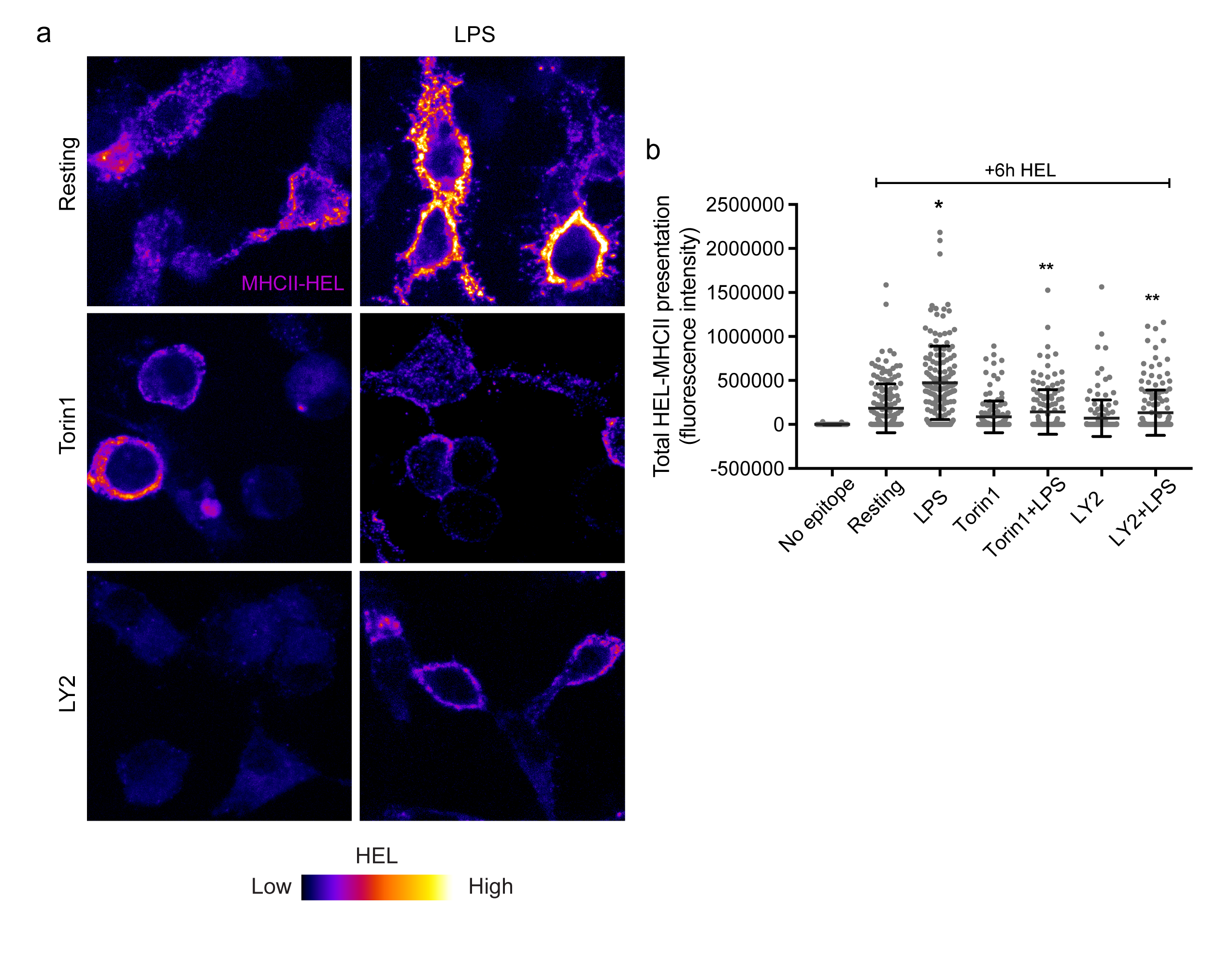

Supplement: S10 Fig — (a) I-Ak::HEL46–61 presentation in BMDCs after incubation with HEL for 6 h in the presence and/or absence of LPS, torin1, and LY2. I-Ak::HEL46–61 cell surface levels were detected by staining unpermeabilized cells with the monoclonal antibody Aw3.18.14. (b) Quantification of total average fluorescence intensity of I-Ak::HEL46–61 complexes at the plasma membrane. Shown is the mean ± SD from 3 experiments, from which 50 to 100 cells were quantified for each. Data were analysed using ANOVA, in which a single asterisk indicates a difference compared with the Resting + HEL condition and two asterisks indicate a difference compared with HEL+LPS (p < 0.05). Scale bar = 15μm. Colour scale: 0 to 12,000 (low to high). See S18 Data for original data in S10 Fig. BMDC, bone marrow–derived dendritic cell; HEL, Hen Egg Lysozyme; LPS, lipopolysaccharides; LY, Lucifer yellow. (TIF) [file pbio.3000535.s010.tif]

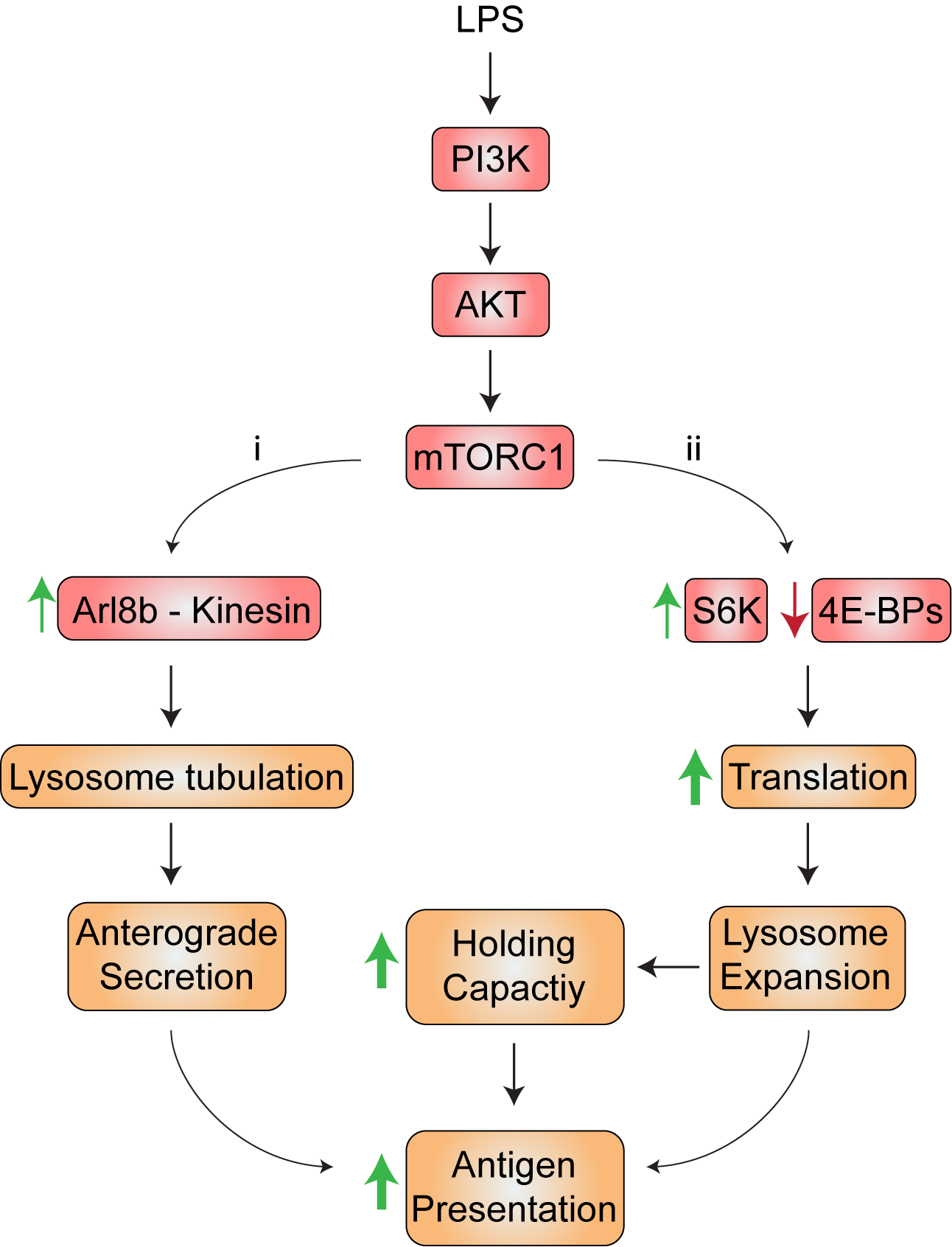

Supplement: S11 Fig — LPS engages the PI3K-AKT-mTOR signal axis to stimulate mTORC1 activity. We suggest that mTORC1 then regulates 2 parallel pathways to modulate lysosome size and morphology: (i) mTORC1 activity augments Arl8b GTPase levels on the lysosome membrane to boost kinesin-1 recruitment to coordinate lysosome extension and anterograde transport. (ii) In parallel, mTORC1 stimulates S6Ks and inhibits 4E-BPs to promote translation and rapidly boost levels of various (select) endo-lysosomal proteins, catalysing endo-lysosome expansion. This expansion increases the holding capacity of the endo-lysosomal system, likely promoting antigen retention. Together both pathways, (i) and (ii), converge to promote lysosome remodelling, collectively bolstering immunity. Importantly, this model does not imply that mTORC1 has no additional functions contributing to phagocyte activation and antigen presentation, nor does it imply that enhanced translation only boosts endo-lysosomal function. Arl8b, ADP Ribosylation Factor Like GTPase 8B; LPS, lipopolysaccharides; mTOR, mechanistic target of rapamycin; mTORC1, mechanistic target of rapamycin complex 1; PI3K, phosphoinositide 3-kinase; S6K, S6 kinase; 4E-BP, 4E-binding protein. (TIF) [file pbio.3000535.s011.tif]
