# Supplementary material for: Enhanced translation expands the endo-lysosome size and promotes antigen presentation during phagocyte activation
Source: PLoS Biol. 2019 Dec 4;17(12):e3000535. doi: 10.1371/journal.pbio.3000535 (PMC6913987; doi:10.1371/journal.pbio.3000535)

As in Figure 2a

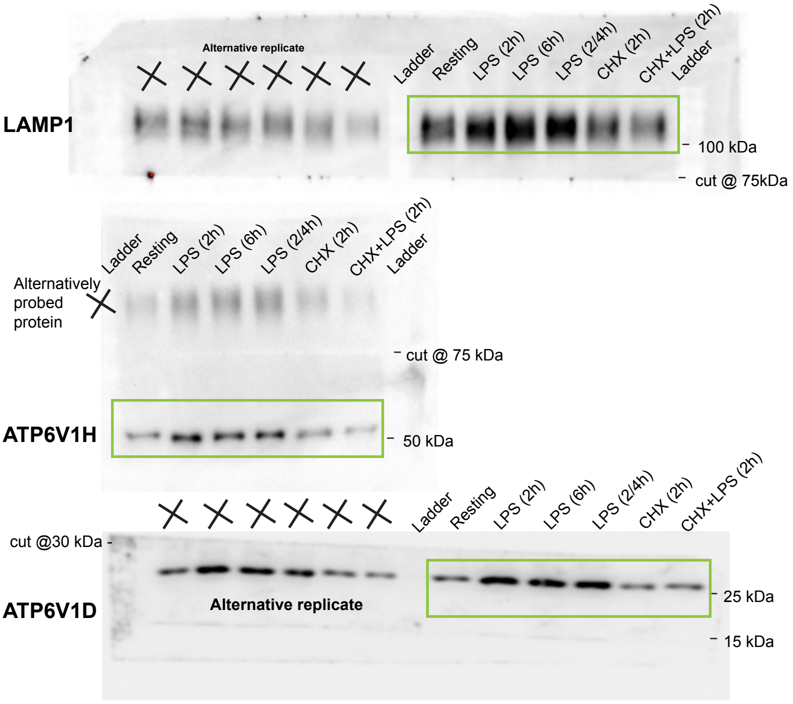

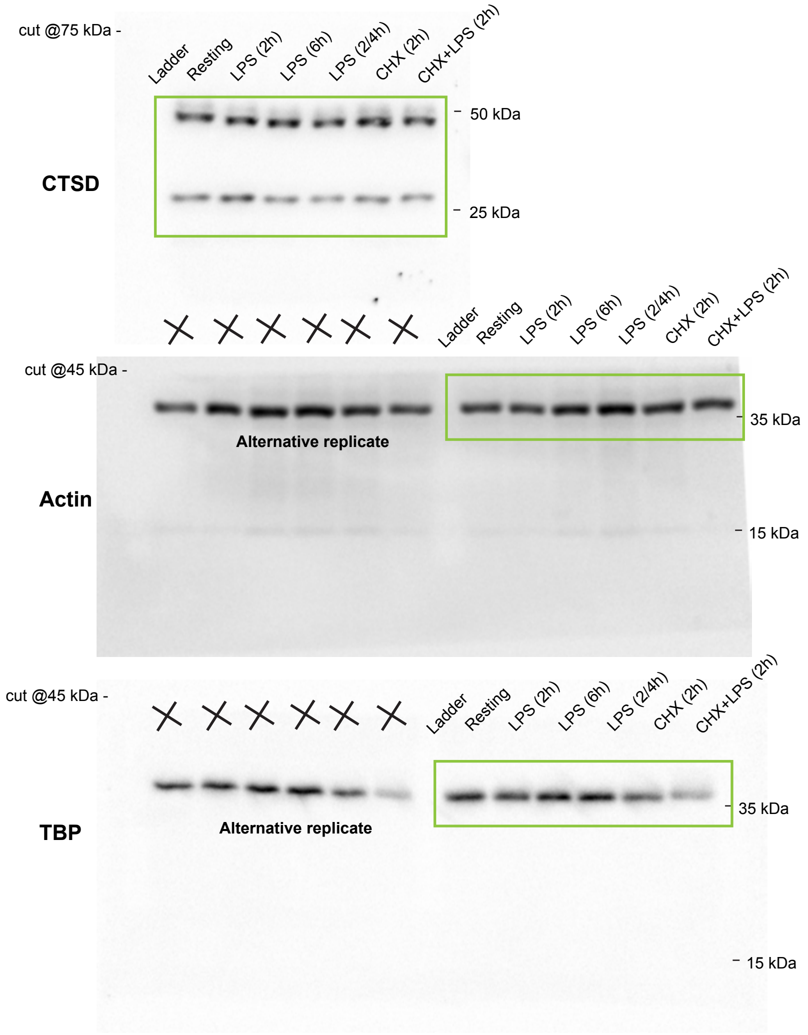

Notes for all original blots and images shown:
*Approx. Molecular weight markers indicated and location where blots were cut to image two separate proteins
*Blots were imaged using chemiluminescence charge-coupled device (CCD) camera–based digital imaging instruments

As in Figure 4c

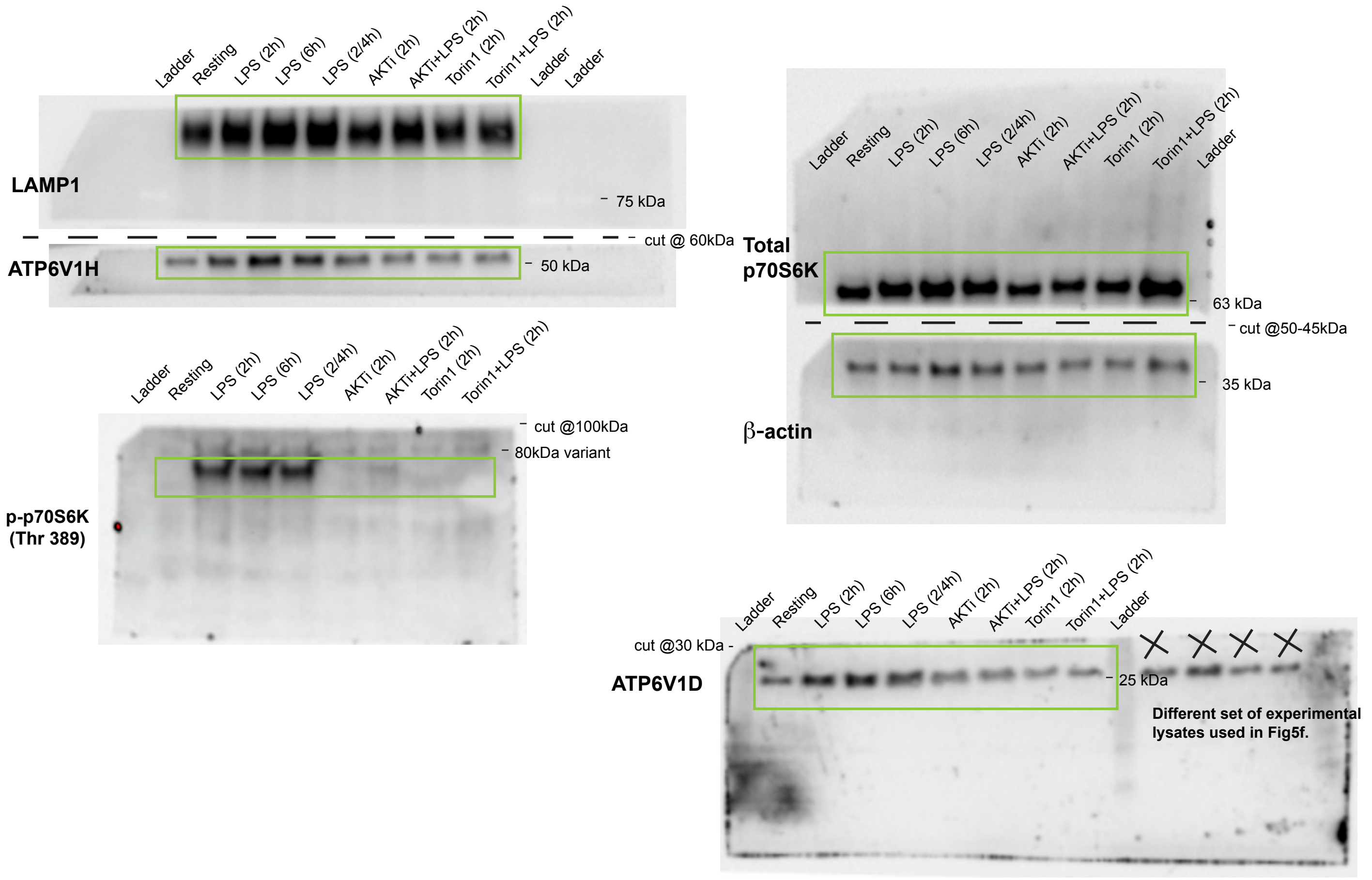

As in Figure 5a

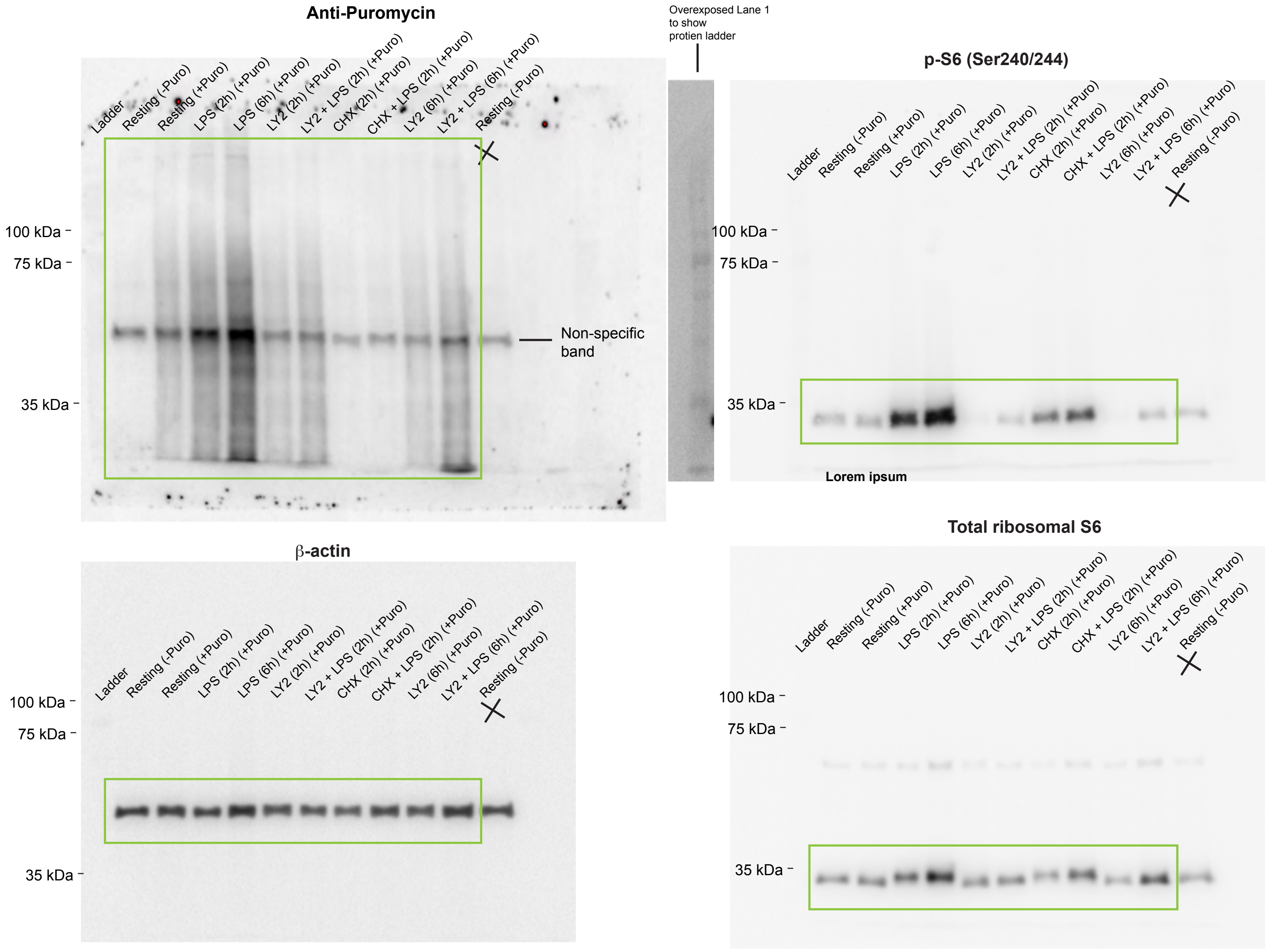

As in Figure 5f

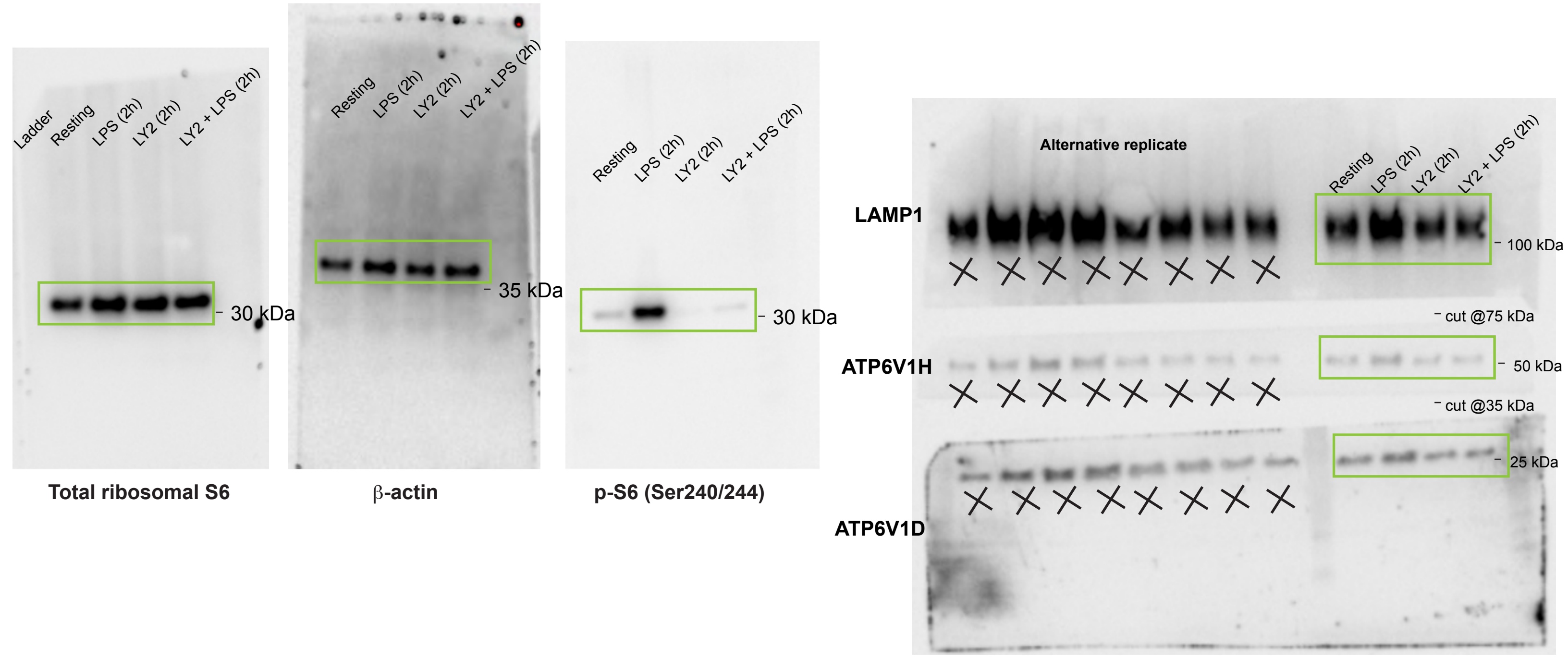

As in Figure 6d

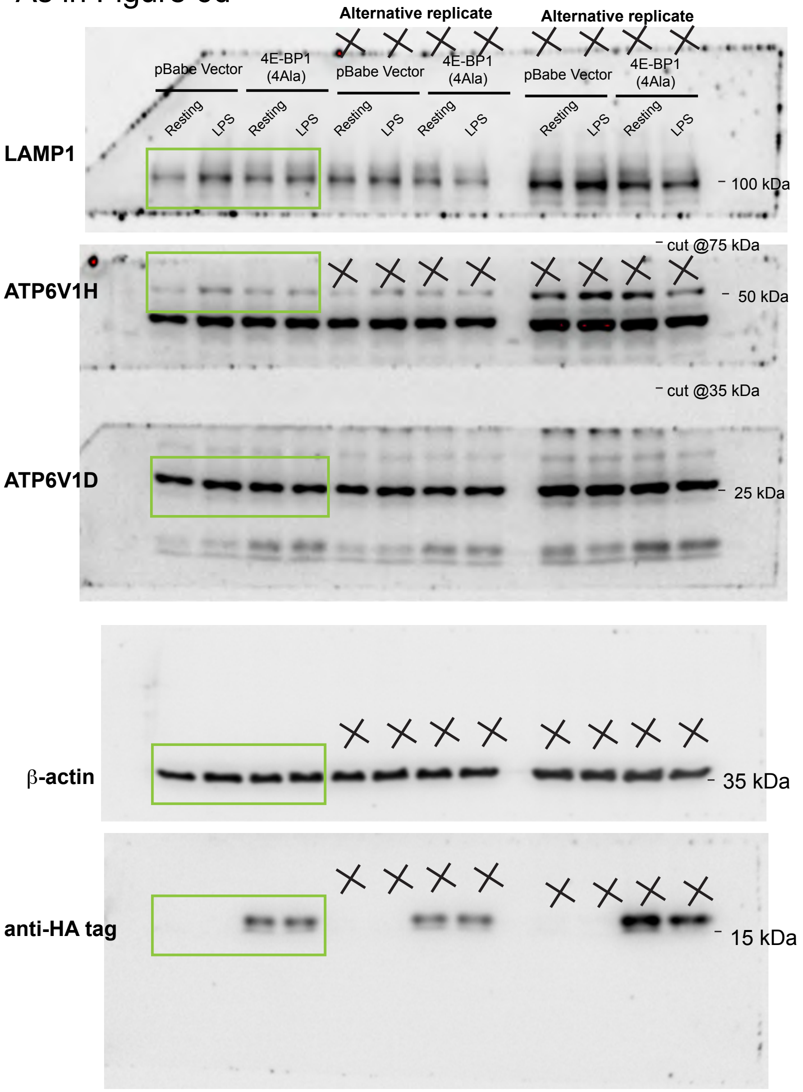

As in Figure 9d

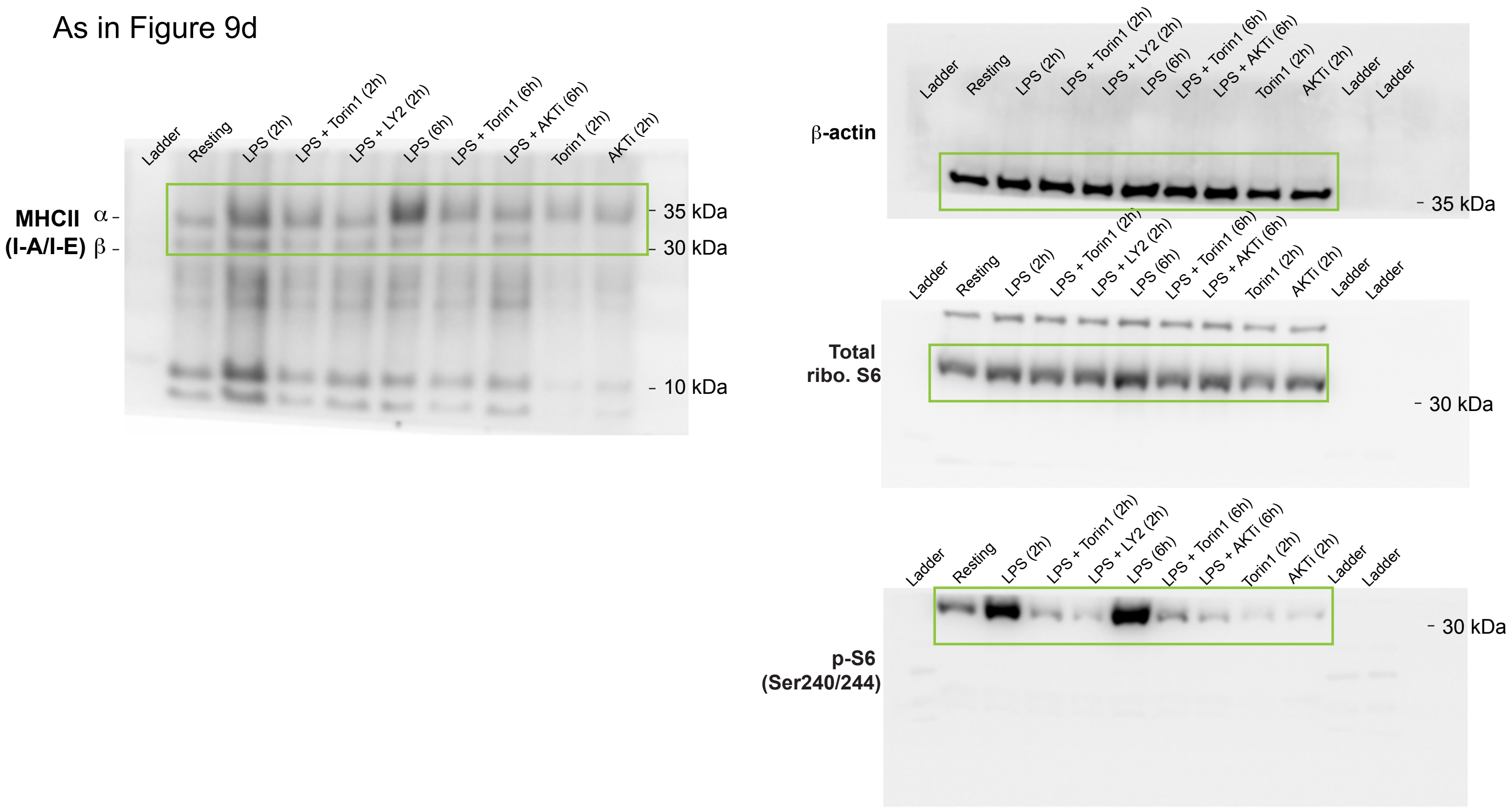

As in Supp_Fig 3a

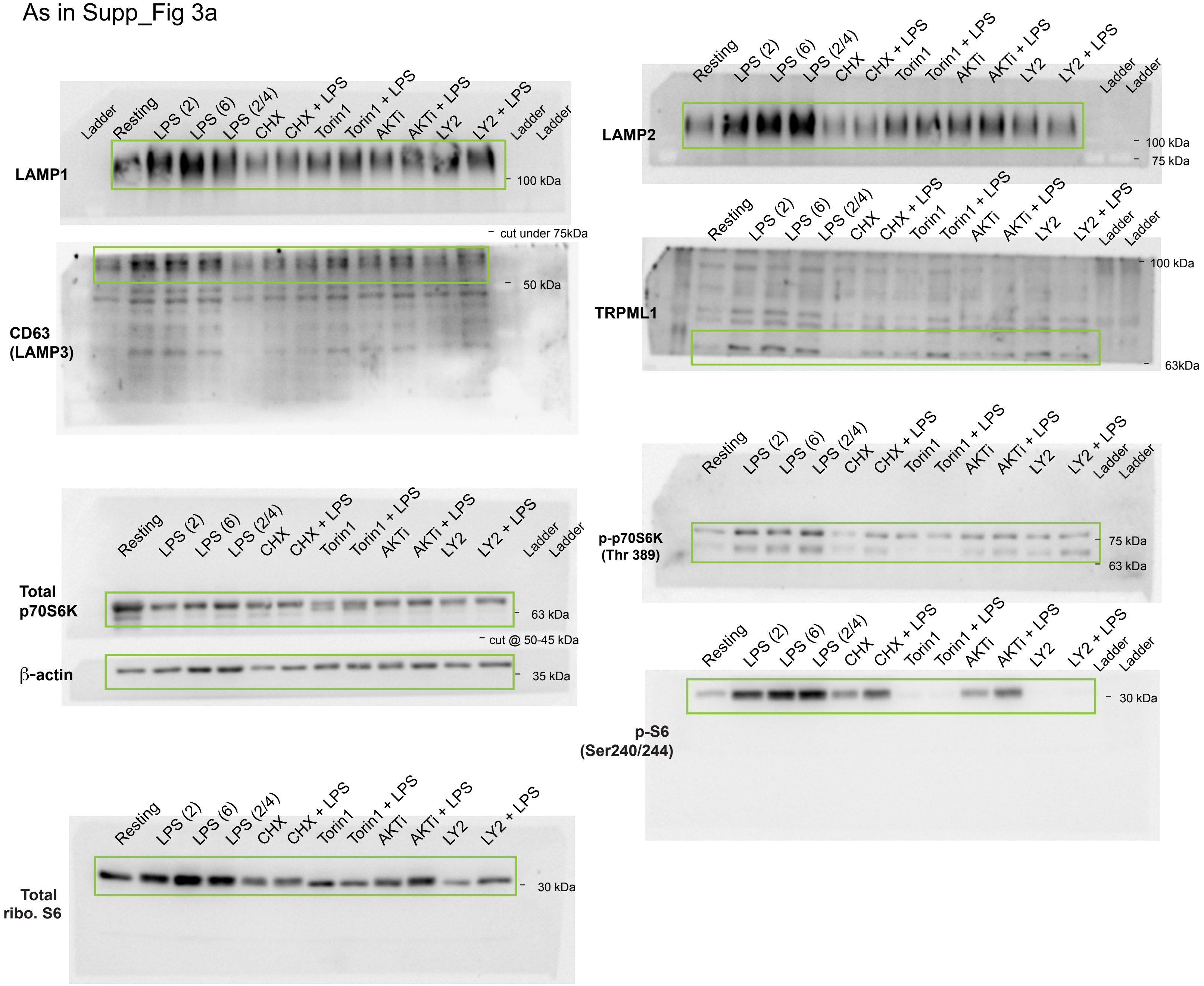

As in Supp_Fig 4a

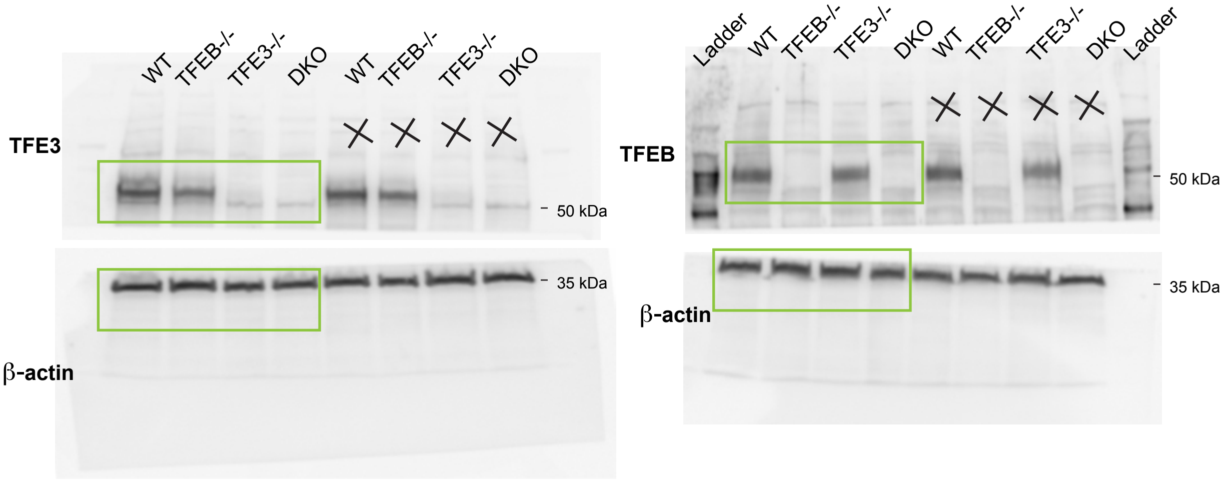

As in Supp_Fig 4a

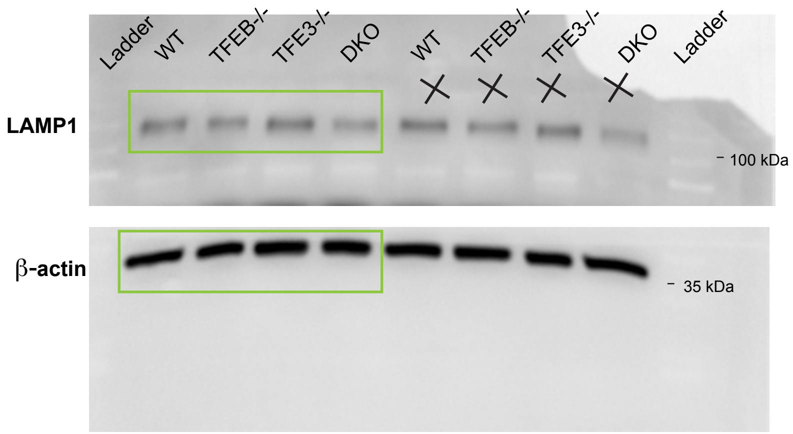

As in Supp_Fig 5a

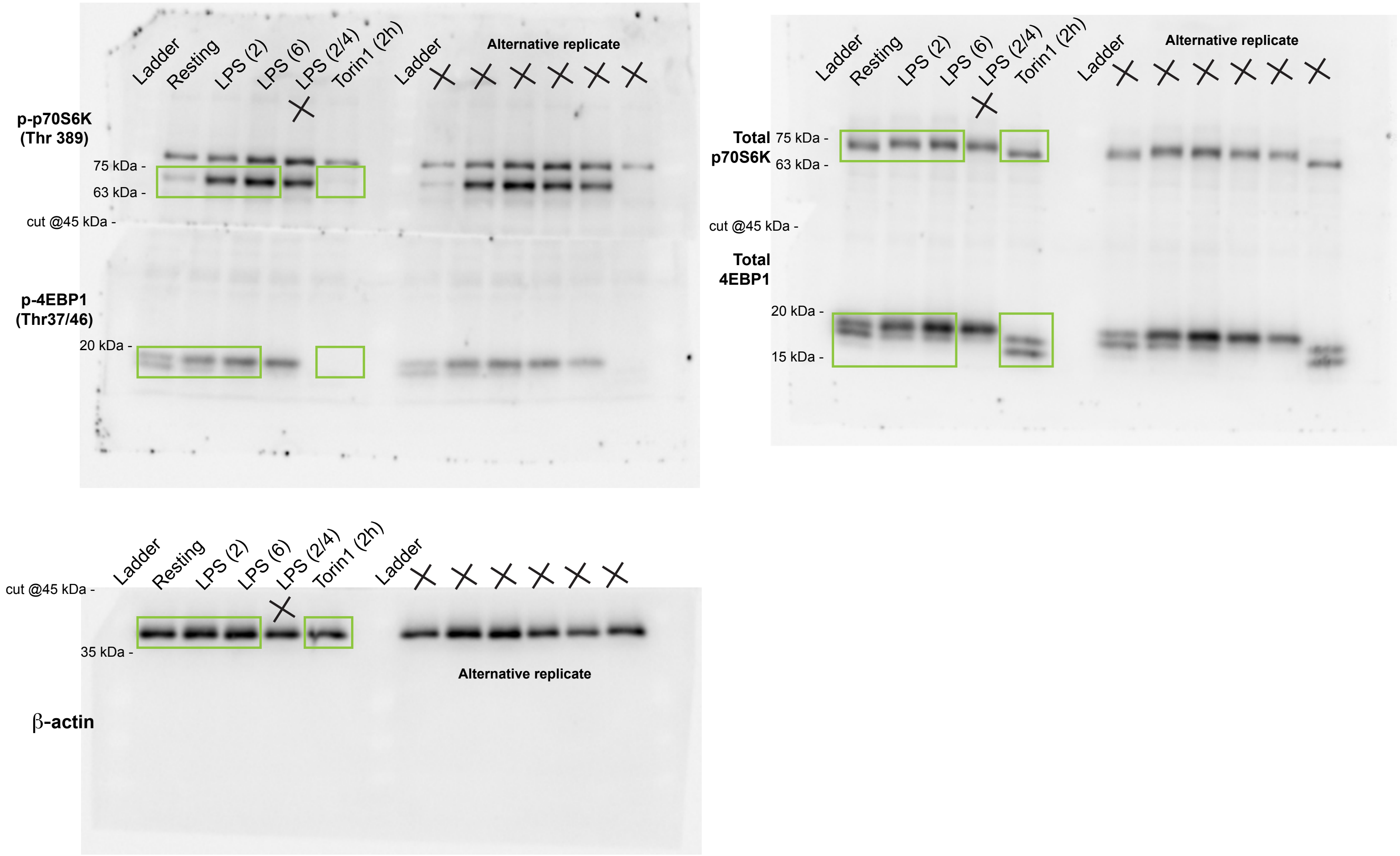

As in Supp_Fig 5d

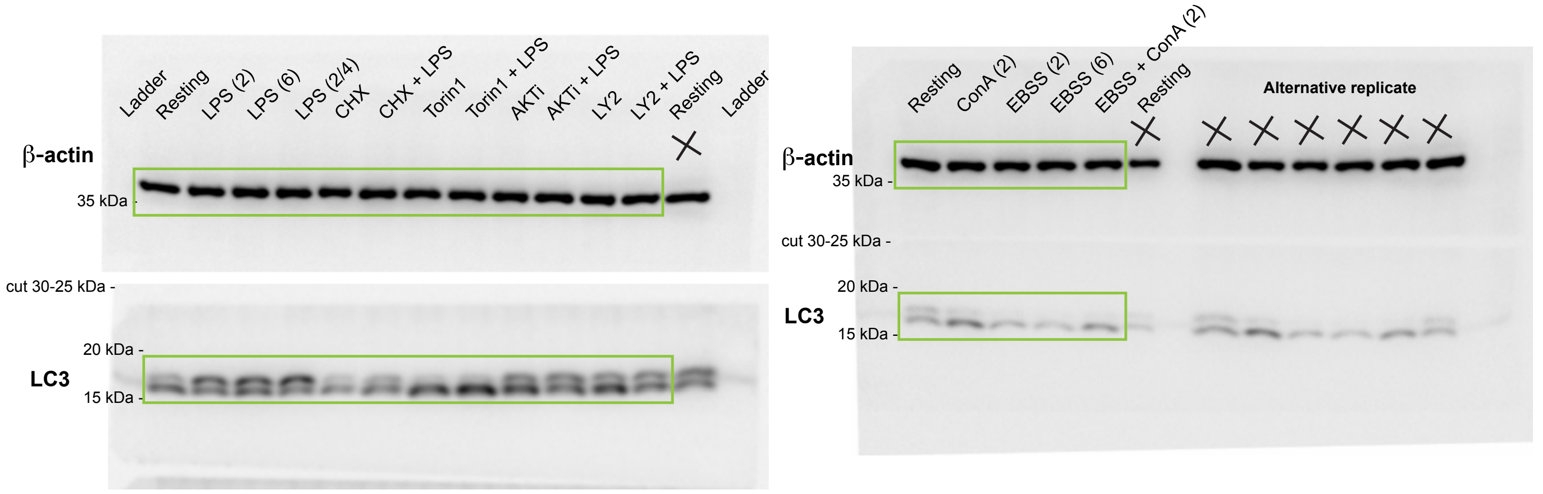

As in Supp_Fig 5f

Anti-Puromycin

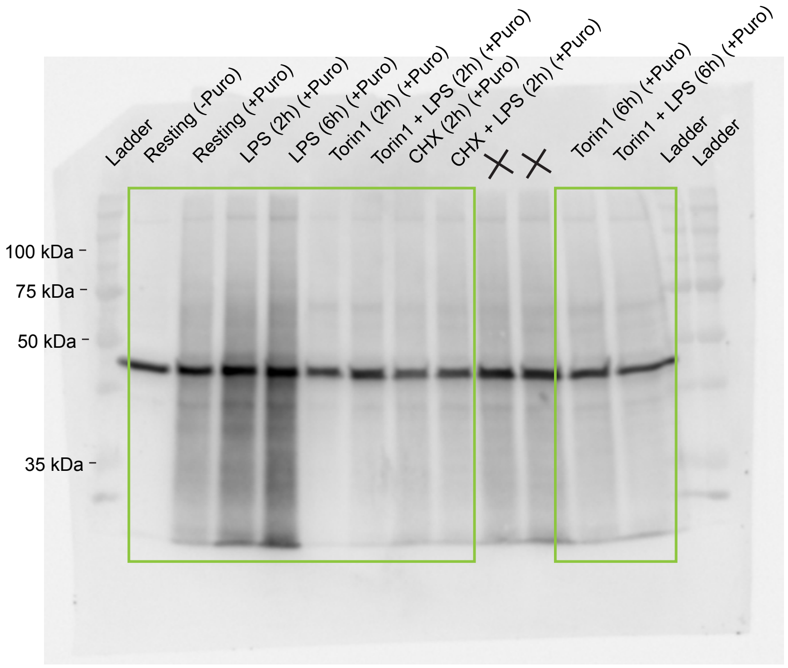

Total p70S6K

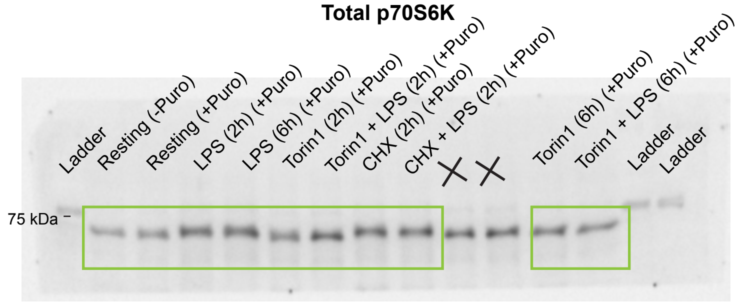

p-p70S6K (Thr 389)

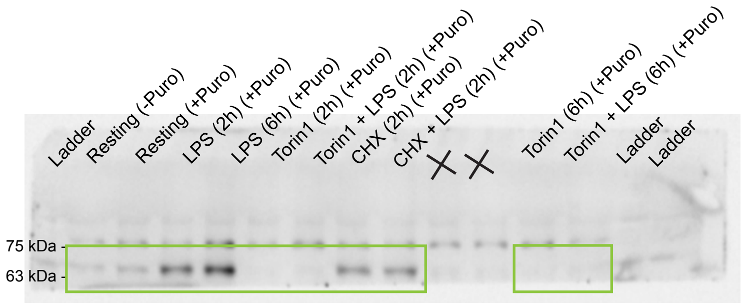

β-actin

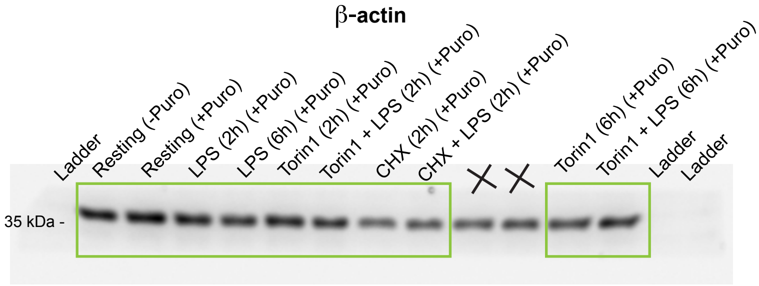

Supplement: S1 Raw Images — (PDF) [file pbio.3000535.s031.pdf]
